# German Veterinary Medical Association from New York and Vicinity

**Published:** 1894-09

**Authors:** H. Wellner

**Affiliations:** Secretary; New York


### SOCIETY PROCEEDINGS.

### GERMAN VETERINARY MEDICAL ASSOCIATION FROM NEW YORK AND VICINITY.

The regular monthly meeting was held Tuesday evening, March 13th, at Dr. Sattler's office, Newark, 112 Boyd Street. The meeting was called to order by the president, Dr. Sattler; upon roll call the following gentlemen responded to their names, viz.: Drs. Turner, Serling. Kuntz, Vogel, Anoker, Patsohek, Wellner, Ogden, Simmons, Hopkins, Weppner, Fraux. The minutes of the previous meeting were read and approved.

Then *Dr. Sattler* read a paper on Hæmorrhagic Nephritis in the horse.

*Dr. Vogel* read an instructive article, "The Anatomy of the Crabfish," and in connection with it he demonstrated two cases of his practice which might be of general interest to the public The first case proves that diphtheria of man is transferable to animals: A private coachman was called to wash a very expensive Angora cat, and his children lately having been sick with diphtheria, playing with said cat caused a disease of this animal This animal died of diphtheria, showing all the pathological symptoms of human diphtheria, which was proved by the post-mortem examination. Another cat of the same family took sick also and died suddenly, and the post-mortem examination showed again the same symptoms. These two cases show plainly the possibility of bacilli of diphtheria causing the same diseases in man as well as in beast. Another case shows that the germs are transferable from beast to man. The children of another family, playing with a parrot who was infected with diphtheria, took the disease of said bird. The above named veterinarian, therefore, advises not to allow children to have sick animals for their playmates.

Another very interesting case was communicated by Dr. Serling, who states that accidentally examining the lungs of calves in a butchers store he found the lungs filled with worms (Strongylus micrurus), which parasites had found their way into the respiratory organs from the stomach through the œsophagus, causing a bronchitis, and, as the result of it, "phthisis pulmonorum," what was proved by Koch feeding cats and dogs with such lungs. Koch calls this disease "Lungenhaarwurmkraukheis" bedingt durth den von ihm so genaunten micrococcus. "Pseudalius ovis pulmonalis auch identisch mit Strongylus rufescens." This proves evidently what a danger the selling of such veals includes for human beings. It was the general opinion of all members present that the Board of Health should investigate such cases which are in a close relation to the welfare of man. Therefore it was the united wish of the Association that the Board of Health, having brought a bill before the legislation regarding pleuro-pneumonia, should also take position towards the question of meat inspection, as this inspection of meat is ruled by laws in very civilized counties. After this subject matter had been discussed, the following resolutions were adopted by the Association, and the secretary directed to transmit them to the respective Board, to whom they were addressed :

Resolution of the German Veterinary Medical Association of New York and Vicinity to the Board of Health of the City of New York:

*Whereas*, The pet animals, as dogs, cats, parrots, etc., are in close communication with human beings, especially with children, in the state of their health as well as their sickness;

*Whereas*, Said animals, like human beings, are subjected to tuberculosis and diphtheria, of which diseases thousands of animals are dying yearly;

*Whereas*, That it is a well known and undeniable fact of these dieases being transferable from human beings to animals and from animals to human beings; and

*Whereas*, That the Committee on Infectious Diseases have reported in our Society meetings of cases in animals having caught these diseases from human beings living in the same houses, and of human beings having caught their diseases from animals;

*Resolved*, That the German Veterinary Medical Association of New York and Vicinity, in calling the attention of the Board of Health of the city of New York to this fact, respectfully recommends, through its President and Secretary,

1st.—That the Board of Health of the city of New York urge every veterinary surgeon of the city of New York to report to said Board such cases when occurring, and

2nd.—That the Board of Health meet every such case according to the respective laws.

After some other business, the meeting adjourned, to meet second Tuesday in April. at Dr. Sattler's office, Newark, 112 Boyd Street.

H. Wellner, Secretary.

New York, March 12th, 1894.

